# Supplementary material for: Systematic Review with Meta-Analyses: Diagnostic Accuracy of FibroMeter Tests in Patients with Non-Alcoholic Fatty Liver Disease
Source: J Clin Med. 2021 Jun 29;10(13):2910. doi: 10.3390/jcm10132910 (PMC8269151; doi:10.3390/jcm10132910)
Supplement: Supplementary file 1 [file jcm-10-02910-s001.zip › jcm-1249967-supplementary.pdf]

Supplementary materials

Supplementary Table 1. PRISMA DTA Checklist

Section/topic	#	PRISMA-DTA Checklist Item	Reported on page #
**TITLE / ABSTRACT**			
Title	1	Identify the report as a systematic review (+/- meta-analysis) of diagnostic test accuracy (DTA) studies.	1
Abstract	2	Abstract: See PRISMA-DTA for abstracts.	1
**INTRODUCTION**			
Rationale	3	Describe the rationale for the review in the context of what is already known.	1-2
Clinical role of index test	D1	State the scientific and clinical background, including the intended use and clinical role of the index test, and if applicable, the rationale for minimally acceptable test accuracy (or minimum difference in accuracy for comparative design).	2
Objectives	4	Provide an explicit statement of question(s) being addressed in terms of participants, index test(s), and target condition(s).	2
**METHODS**			
Protocol and registration	5	Indicate if a review protocol exists, if and where it can be accessed (e.g., Web address), and, if available, provide registration information including registration number.	3
Eligibility criteria	6	Specify study characteristics (participants, setting, index test(s), reference standard(s), target condition(s), and study design) and report characteristics (e.g., years considered, language, publication status) used as criteria for eligibility, giving rationale.	3
Information sources	7	Describe all information sources (e.g., databases with dates of coverage, contact with study authors to identify additional studies) in the search and date last searched.	3
Search	8	Present full search strategies for all electronic databases and other sources searched, including any limits used, such that they could be repeated.	3
Study selection	9	State the process for selecting studies (i.e., screening, eligibility, included in systematic review, and, if applicable, included in the meta-analysis).	4
Data collection process	10	Describe method of data extraction from reports (e.g., piloted forms, independently, in duplicate) and any processes for obtaining and confirming data from investigators.	4
Definitions for data extraction	11	Provide definitions used in data extraction and classifications of target condition(s), index test(s), reference standard(s) and other characteristics (e.g. study design, clinical setting).	4
Risk of bias and applicability	12	Describe methods used for assessing risk of bias in individual studies and concerns regarding the applicability to the review question.	4
Diagnostic accuracy measures	13	State the principal diagnostic accuracy measure(s) reported (e.g. sensitivity, specificity) and state the unit of assessment (e.g. per-patient, per-lesion).	3
Synthesis of results	14	Describe methods of handling data, combining results of studies and describing variability between studies. This could include, but is not limited to: a) handling of multiple definitions of target condition. b)	3-4

		handling of multiple thresholds of test positivity, c) handling multiple index test readers, d) handling of indeterminate test results, e) grouping and comparing tests, f) handling of different reference standards	

Section/topic	#	PRISMA-DTA Checklist Item	Reported on page #
Meta-analysis	D2	Report the statistical methods used for meta-analyses, if performed.	4-5
Additional analyses	16	Describe methods of additional analyses (e.g., sensitivity or subgroup analyses, meta-regression), if done, indicating which were pre-specified.	4-5
**RESULTS**			
Study selection	17	Provide numbers of studies screened, assessed for eligibility, included in the review (and included in meta-analysis, if applicable) with reasons for exclusions at each stage, ideally with a flow diagram.	5, Figure 1
Study characteristics	18	For each included study provide citations and present key characteristics including: a) participant characteristics (presentation, prior testing), b) clinical setting, c) study design, d) target condition definition, e) index test, f) reference standard, g) sample size, h) funding sources	6-7
Risk of bias and applicability	19	Present evaluation of risk of bias and concerns regarding applicability for each study.	9
Results of individual studies	20	For each analysis in each study (e.g. unique combination of index test, reference standard, and positivity threshold) report 2x2 data (TP, FP, FN, TN) with estimates of diagnostic accuracy and confidence intervals, ideally with a forest or receiver operator characteristic (ROC) plot.	10-14
Synthesis of results	21	Describe test accuracy, including variability; if meta-analysis was done, include results and confidence intervals.	10-14
Additional analysis	23	Give results of additional analyses, if done (e.g., sensitivity or subgroup analyses, meta-regression; analysis of index test: failure rates, proportion of inconclusive results, adverse events).	14
**DISCUSSION**			
Summary of evidence	24	Summarize the main findings including the strength of evidence.	15
Limitations	25	Discuss limitations from included studies (e.g. risk of bias and concerns regarding applicability) and from the review process (e.g. incomplete retrieval of identified research).	16
Conclusions	26	Provide a general interpretation of the results in the context of other evidence. Discuss implications for future research and clinical practice (e.g. the intended use and clinical role of the index test).	16-17
**FUNDING**			
Funding	27	For the systematic review, describe the sources of funding and other support and the role of the funders.	17

Adapted From: McInnes MDF, Moher D, Thombs BD, McGrath TA, Bossuyt PM, The PRISMA-DTA Group (2018). Preferred Reporting Items for a Systematic Review and Meta-analysis of Diagnostic Test Accuracy Studies: The PRISMA-DTA Statement. JAMA. 2018 Jan 23;319(4):388-396. doi: 10.1001/jama.2017.19163.

Supplementary Table 2. OVID via MEDLINE search strategy, search tables for other databases were similar (not presented)

1	exp Fatty Liver/	31239
2	(NAFL* or NASH*).mp.	19808
3	"non-alcoholic fatty liver disease*".mp.	15260
4	(((fatty or fat or steato*) adj3 (liver* or hepat*)) or steatohepat* or (visceral adj2 steato*)).ti,ab.	48508
5	1 or 2 or 3 or 4	60354
6	exp "sensitivity and specificity"/ or exp "mass screening"/ or "reference values"/ or "false positive reactions"/ or "false negative reactions"/ or specificit$.tw. or screening.tw. or false positive$.tw. or false negative$.tw. or accuracy.tw. or predictive value$.tw. or reference value$.tw. or roc$.tw. or likelihood ratio$.tw. or predictive value$.tw.	1925738
7	(fibro meter* or fibrometer or fibro-meter*).ti,ab,kf	114
8	5 and 6 and 7	15
9	exp animals/ not humans/	4648880
10	8 not 9	15
11	limit 10 to yr="2019 -Current"	4

Supplementary Table 3. Correspondence between the NASH CRN and the METAVIR systems. Reported by Boursier et al. 2017[22]

**Metavir**	**NASH CRN**	**Present study**
F0	F0 or F1 (isolated perisinusoidal fibrosis)	No/mild fibrosis
F1	F1 (isolated periportal fibrosis) or F2	≥F2 significant fibrosis
F2	F3	≥F3 advanced fibrosis
F3	F3	
F4	F4	

Supplementary Table 4. Liver biopsy characteristics.

	Study ID	Mean age (years)	Needle gauge (mm)	Biopsy length (mm)	Portal tracts	Time interval	Pathologist	Blinded to other tests
1	Aykut 2014[17]	46	16	≥ 20*	≥11	NR	Single pathologist	NR
2	Boursier 2016[13]	55.9	NR	27 (11)	NR	< 1 week	Single hepatopatologist	Yes
3	Boursier 2019[26]	56.5	NR	27(12) #	NR	< 1 week	Single hepatopatologist (3)	Yes
4	Cales 2009[27]	51.1	14-15	NR	NR	< 3 months (4)	One or two pathologists	NR
5	Dincses 2015[29]	45	16	20*	11	NR	Single pathologist	NR
6	Eddowes 2017[15]	36-65 (79.3%) >65 (21%)	NR	NR	NR	NR	Two expert pathologists	Yes
7	Loong 2017[29]	52	16	NR	NR	<1 day	Two experienced pathologist	Yes
8	Sanyal 2016[34]	52.1	NR	NR	NR	NR	Centrally read by single pathologist	Yes
9	Siddiqui 2016[30]	52.9	NR	NR	NR	<2 months	Single hepatopathologist	Yes
10	Staufer 2019[31]	52	NR	> 15mm; mean 22mm	NR	<1 day	Two experienced hepatopathologists	Yes
11	Subasi 2015[18]	45	NR	≥ 20*	11	NR	NR	NR
12	Yang 2019[32]	36.6	16	>15mm	>5	<1 week	Reevaluated by two experienced pathologists	Yes

* All liver biopsy specimens were at least 20 mm long and/or contained more than 11 complete portal tracts; (3) Pathological examinations were performed in each center by the same senior expert specialized in hepatology and blinded to patient data; # 89.0% of the liver biopsies were ≥15 mm in length; (4) Liver biopsy generally within one week (maximum 3 months);
NR: not reported

Supplementary Table 5. Number of included studies in three different meta-analyses all with target condition advanced fibrosis (F≥3)

FibroMeter	No. of studies included	Patients included	Patients with advanced fibrosis	
V2G	3	1576	604	Boursier 2016; Boursier 2019; Staufer 2019
NAFLD	7	1616	514	Aykut 2014; Boursier 2016; Loong 2017; Siddiqui 2016; Subasi 2015; Yang 2019; Cales 2009
VCTE	4	1546	542	Dincses 2015; Eddowes 2017; Loong 2017; Boursier 2019

Supplementary Table 6. Summary of accuracy of FibroMeter VCTE, V2G and NAFLD for advanced fibrosis, presented in AUC, sensitivity and specificity. Including results after sensitivity analysis.

FibroMeter version	No. of studies included	AUC	Sensitivity (95% CI)	Specificity (95% CI)
FM V2G	3	0.89	0.83 (0.73- 0.90)	0.84 (0.62- 0.95)
FM NAFLD	7	0.82	0.65 (0.51- 0.77)	0.86 (0.75- 0.93)
FM VCTE	4	0.94	0.70 (0.33- 0.92)	0.93 (0.88- 0.96)
Sensitivity analysis				
FM NAFLD	6	0.79	0.72 (0.63-0.79)	0.828 (0.71-0.91)
FM VCTE	3	0.92	0.84 (0.58- 0.94)	0.911 (0.89-0.93)

AUC = area under the curve; 95%CI = 95% confidence interval

Supplementary Figure 1. Graphical summary of the methodological quality of included studies using the QUADAS-2 tool

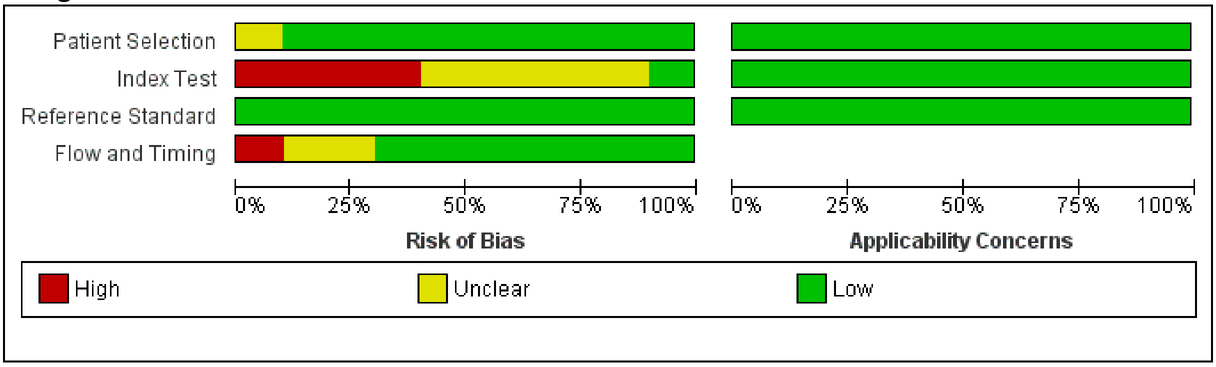

Supplementary Figure 2. Methodological quality of each of the included studies per domain of the QUADAS-2 tool

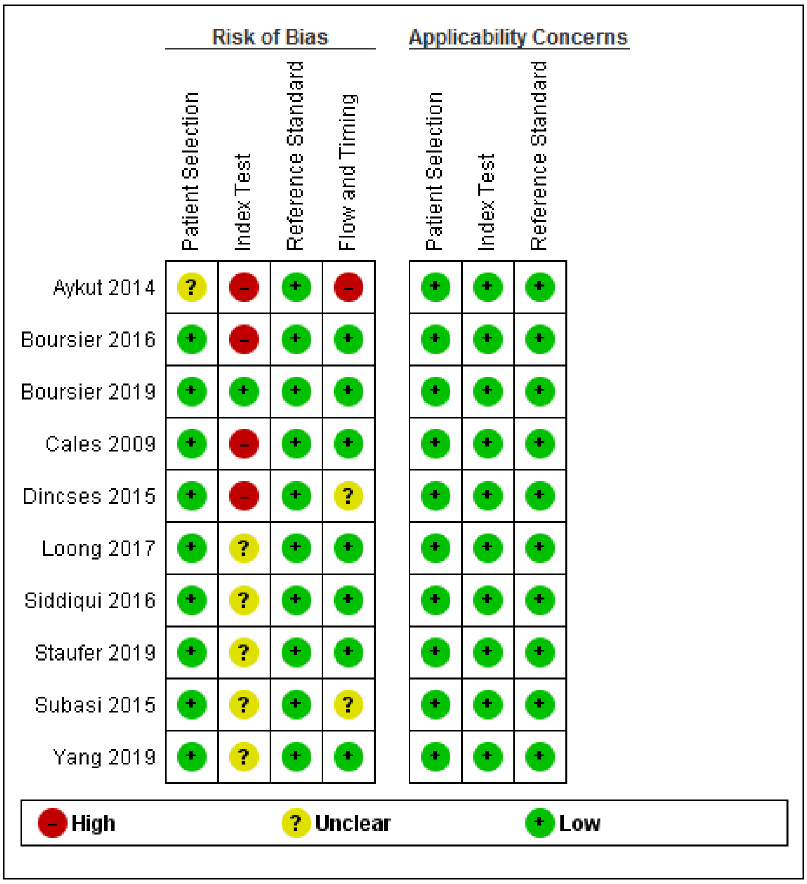

Supplementary Figure 3. Forest plot FibroMeter V2G

Study	TP	FP	FN	TN	Cut-off	Prevalence	Sensitivity (95% CI)	Specificity (95% CI)
Boursier 2016	132	79	40	201	0.45	0.38	0.77 [0.70, 0.83]	0.72 [0.66, 0.77]
Boursier 2019	341	35	43	519	0.77	0.41	0.89 [0.85, 0.92]	0.94 [0.91, 0.96]
Staufer 2019	39	16	9	67	0.39	0.37	0.81 [0.67, 0.91]	0.81 [0.71, 0.89]

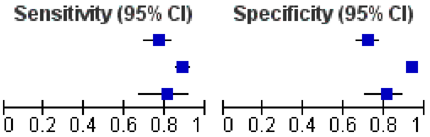

Supplementary Figure 4. Forest plot FibroMeter NAFLD

Study	TP	FP	FN	TN	Cut-off	Prevalence	Sensitivity (95% CI)	Specificity (95% CI)
Aykut 2014	18	17	9	44	0.82	0.31	0.67 [0.46, 0.83]	0.72 [0.59, 0.83]
Boursier 2016	137	107	35	173	0.31	0.38	0.80 [0.73, 0.85]	0.62 [0.56, 0.68]
Cales 2009	52	2	14	167	0.49	0.28	0.79 [0.67, 0.88]	0.99 [0.96, 1.00]
Loong 2017	9	6	34	166	0.82	0.2	0.21 [0.10, 0.36]	0.97 [0.93, 0.99]
Siddiqui 2016a	46	34	5	60	0.33	0.35	0.90 [0.79, 0.97]	0.64 [0.53, 0.73]
Siddiqui 2016b	38	13	13	81	0.59	0.35	0.75 [0.60, 0.86]	0.86 [0.78, 0.92]
Siddiqui 2016c	28	9	23	85	0.83	0.35	0.55 [0.40, 0.69]	0.90 [0.83, 0.96]
Subasi 2015	20	29	10	83	0.82	0.21	0.67 [0.47, 0.83]	0.74 [0.65, 0.82]
Yang 2019	75	44	51	283	0.72	0.28	0.60 [0.50, 0.68]	0.87 [0.82, 0.90]

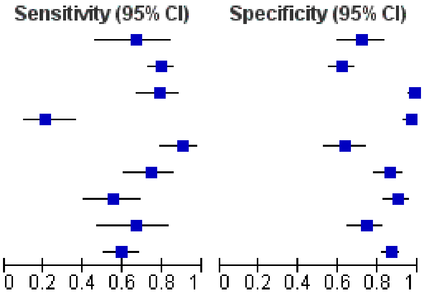

Supplementary Figure 5. Forest plot FibroMeter VCTE

Study	TP	FP	FN	TN	Cut-off	Prevalence	Sensitivity (95% CI)	Specificity (95% CI)
Boursier 2019	347	47	37	507	0.69	0.41	0.90 [0.87, 0.93]	0.92 [0.89, 0.94]
Dincses 2015	9	3	1	39	0.8	0.19	0.90 [0.55, 1.00]	0.93 [0.81, 0.99]
Eddowes 2017a	95	69	11	128	0.38	0.35	0.90 [0.82, 0.95]	0.65 [0.58, 0.72]
Eddowes 2017b	84	33	22	164	0.55	0.35	0.79 [0.70, 0.87]	0.83 [0.77, 0.88]
Eddowes 2017c	71	20	35	177	0.67	0.35	0.67 [0.57, 0.76]	0.90 [0.85, 0.94]
Loong 2017	8	1	35	171	0.8	0.2	0.19 [0.08, 0.33]	0.99 [0.97, 1.00]

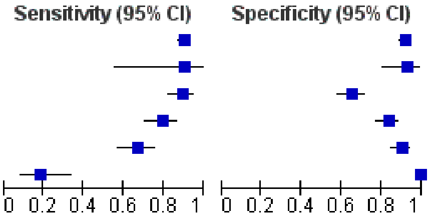

References

22. Boursier J, de Ledinghen V, Leroy V, et al. A stepwise algorithm using an at-a-glance first-line test for the non-invasive diagnosis of advanced liver fibrosis and cirrhosis. *J Hepatol*. 2017;66(6):1158-1165. doi:10.1016/j.jhep.2017.01.003

17. Aykut UE, Akyuz U, Yesil A, et al. A comparison of fibrometer^{TM} NAFLD score, NAFLD fibrosis score, and transient elastography as noninvasive diagnostic tools for hepatic fibrosis in patients with biopsy-proven non-alcoholic fatty liver disease. *Scand J Gastroenterol*. 2014;49(11):1343-1348. doi:10.3109/00365521.2014.958099

13. Boursier J, Vergniol J, Guillet A, et al. Diagnostic accuracy and prognostic significance of blood fibrosis tests and liver stiffness measurement by FibroScan in non-alcoholic fatty liver disease. *J Hepatol*. 2016;65(3):570-578. doi:10.1016/j.jhep.2016.04.023

26. Boursier J, Guillaume M, Leroy V, et al. New sequential combinations of non-invasive fibrosis tests provide an accurate diagnosis of advanced fibrosis in NAFLD. *J Hepatol*. 2019;71(2):389-396. doi:10.1016/j.jhep.2019.04.020

27. Calès P, Lainé F, Boursier J, et al. Comparison of blood tests for liver fibrosis specific or not to NAFLD. *J Hepatol*. 2009;50(1):165-173. doi:10.1016/j.jhep.2008.07.035

29. Dincses E, Yilmaz Y. Diagnostic usefulness of FibroMeter VCTE for hepatic fibrosis in patients with nonalcoholic fatty liver disease. *Eur J Gastroenterol Hepatol*. 2015;27(10):1149-1153. doi:10.1097/MEG.0000000000000409

15. Peter J. Eddowes, Michael E. Allison, Emmanuel Tsochatzis, Quentin M. Anstee, Indra Neil Guha, Jeremy F. Cobbold, David A. Sheridan, Valerie Paradis, Pierre Bedossa PN. Comparison of published noninvasive biomarkers to reliably exclude severe fibrosis in NAFLD patients. 2017;(October).

29. Loong TCW, Wei JL, Leung JCF, et al. Application of the combined FibroMeter vibration-controlled transient elastography algorithm in Chinese patients with non-alcoholic fatty liver disease. *J Gastroenterol Hepatol*. 2017. doi:10.1111/jgh.13671

34. A.J. S, S.A. H, G. C, et al. Assessment of serum levels of Chitinase-3-like protein 1 (CHI3L1) improves identification of the NASH patients at risk who should be treated. *Hepatology*. 2016.

30. Siddiqui MS, Patidar KR, Boyett S, Luketic VA, Puri P, Sanyal AJ. Performance of non-invasive models of fibrosis in predicting mild to moderate fibrosis in patients with non-alcoholic fatty liver disease. *Liver Int*. 2016. doi:10.1111/liv.13054

31. Staufer K, Halilbasic E, Spindelboeck W, et al. Evaluation and comparison of six noninvasive tests for prediction of significant or advanced fibrosis in nonalcoholic fatty liver disease. *United Eur Gastroenterol J*. 2019. doi:10.1177/2050640619865133

18. Subasi CF, Aykut UE, Yilmaz Y. Comparison of noninvasive scores for the detection of advanced fibrosis in patients with nonalcoholic fatty liver disease. *Eur J Gastroenterol Hepatol*. 2015. doi:10.1097/MEG.0000000000000255

32. Yang M, Jiang L, Wang Y, et al. Step layered combination of noninvasive fibrosis models improves diagnostic accuracy of advanced fibrosis in nonalcoholic fatty liver disease. *J Gastrointest Liver Dis*. 2019. doi:10.15403/jgld-420
